# The TACOS Technique: A Stepwise Protocol for Alveolar Ridge Augmentation Using Customized Titanium Mesh

**DOI:** 10.3390/medicina61010058

**Published:** 2025-01-02

**Authors:** Mauro Merli, Luca Aquilanti, Marco Merli, Giorgia Mariotti, Giorgio Rappelli

**Affiliations:** 1 Department of Clinical Specialistic and Dental Sciences, Polytechnic University of Marche, Via Tronto, 10/A, 60126 Ancona, Italy; mauromerli@gmail.com (M.M.); g.rappelli@staff.univpm.it (G.R.); 2 Independent Researcher, Viale Settembrini, 17/O, 47923 Rimini, Italy; marco.merli5@icloud.com (M.M.); mariottigiorgia@gmail.com (G.M.); 3 Dentistry Clinic, National Institute of Health and Science of Aging, IRCCS INRCA, Via Tronto, 10/A, 60126 Ancona, Italy

**Keywords:** alveolar ridge augmentation, customized titanium mesh, bone grafting, dental implants, bone resorption, guided bone regeneration

## Abstract

*Background:* Alveolar ridge resorption following tooth loss poses a significant challenge for successful dental implant placement. In cases of severe atrophy, bone augmentation is required to restore sufficient bone volume. This technical note outlines a detailed, stepwise surgical protocol for horizontal and vertical alveolar ridge augmentation using customized titanium mesh. *Materials and Methods:* The procedure includes precise mesh fitting, autologous bone grafting, and the application of bioactive agents to promote bone regeneration. Emphasis is placed on the technique’s feasibility, predictability, and the critical steps necessary for preventing complications. *Results:* The use of customized mesh ensures stability and improved bone regeneration outcomes, enabling clinicians to achieve successful implant placement even in severely atrophic ridges. *Conclusions:* The described protocol has demonstrated predictable results in both clinical and radiographic evaluations, offering an effective solution for complex bone augmentation cases.

## 1. Introduction

Tooth or implant loss commonly leads to significant alveolar ridge atrophy, characterized by a reduction in both bone height and width [1]. These changes present challenges in dental implantology, particularly in cases where the residual bone volume is insufficient for implant placement according to prosthetic standards. Although short implants have demonstrated effectiveness in some atrophic jaws, their application is often limited by unfavorable bone anatomy or prosthetic requirements. Consequently, restoring sufficient bone volume is a prerequisite for ensuring stable and predictable implant placement [2,3].

Bone augmentation techniques, including guided bone regeneration (GBR), have emerged as indispensable tools for managing complex defects [4]. GBR involves the use of barrier membranes, either resorbable [5] or non-resorbable, in conjunction with autologous bone grafts or biomaterials [6]. The selection of the membrane type and graft material depends on the defect’s size, shape, and complexity. Non-resorbable membranes have demonstrated superior space-maintaining capacity, making them particularly suitable for vertical and horizontal augmentation [7]. However, these techniques are technically demanding and associated with a higher complication rate, emphasizing the need for advanced surgical expertise.

In recent years, advancements in digital technology and material science have introduced a paradigm shift in GBR procedures. Customized titanium meshes, fabricated using computer-aided design and manufacturing (CAD/CAM) and selective laser melting (SLM), represent a significant innovation in alveolar ridge reconstruction. These meshes are tailored to the patient’s unique anatomy, enabling precise defect coverage, improved graft stability, and predictable outcomes. Compared to traditional non-resorbable membranes, customized titanium meshes offer enhanced mechanical properties, superior defect adaptation, and the ability to pre-plan graft volumes during the diagnostic phase [8].

Despite these advantages, the application of customized titanium meshes requires meticulous surgical planning and execution to minimize complications such as mesh exposure or soft tissue dehiscence. Proper flap design, tension-free closure, and soft tissue management are integral to the success of this approach. Additionally, the integration of bioactive agents has further enhanced the regenerative potential of GBR, accelerating healing and improving soft tissue outcomes.

This paper aims to provide a detailed, stepwise surgical protocol for horizontal and vertical ridge augmentation using customized titanium meshes. The proposed technique, referred to as the TACOS Technique (Titanium Alveolar Customized Osteogenic Scaffold), offers a systematic approach for addressing severe alveolar ridge defects in partially or fully edentulous patients. Unlike previous studies, which have primarily focused on outcomes, this paper emphasizes the practical execution of each surgical step, serving as a comprehensive guide for clinicians. The TACOS Technique underscores the importance of patient-specific solutions in modern implantology and highlights the potential for future innovations in guided bone regeneration.

## 2. Materials and Methods

This procedure is suitable for patients in need of implant treatment and with severe atrophic edentulous alveolar crest in either the upper or the lower jaw. Preoperative assessment includes cone beam computerized tomography (CBCT) imaging to evaluate bone loss and guide the design of a customized titanium mesh. Subjects with systemic or local diseases contraindicating surgical therapy, smokers, and pregnant and lactating women should be excluded.

All patients should undergo conscious sedation with continuous monitoring of vital signs throughout the entire surgery. During the surgical procedures, the patients should receive the following pharmacologic protocol: fractioned administration of midazolam (0.5 to 1 mg) and atropine (0.5 mg), ceftriaxone (1 g), tramadol (100 mg), ketorolac (30 mg), and betamethasone sodium phosphate (4 mg). It is worth noting that the use of these drugs should be personalized accordingly to the characteristics of each patient. The familiarization with the anamnesis of each patient is crucial, as well as a deep knowledge of the active principles of the drugs and their pharmacological activity.

The study was conducted in full accordance with ethical principles, including the Declaration of Helsinki. Written informed consent was obtained from the patient.

### Surgical Protocol: The TACOS Technique

**Step 1: Incision Design and Flap Management**

After local anesthesia (articaine with adrenaline 1:100,000), a mid-crestal in the lower jaw or paracrestal buccal incision in the upper jaw (to anticipate palatal flap elongation) is made at the edentolous ridge site, extending mesially and distally for adequate exposure. The flap management implies a mucogingival approach, which involves the elevation of split thickness surgical papillae, anticipating the coronally advanced flap, before raising a full-thickness flap to expose the whole defect [9].

**Step 2: Site Preparation**

**Figure 1:**
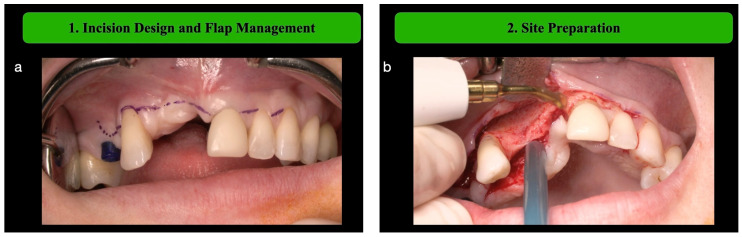
(**a**) Incision design and flap elevation using a 15-C blade and a mucoperiosteal elevator. (**b**) the residual bone crest is completely free from soft tissue.

The recipient site is debrided eliminating all the soft tissue on the crest using manual devices such as little Lucas or Volkmann sharp spoons, or specific piezoelectric ultrasonic inserts (Mectron Piezosurgery, Mectron s.p.a., Carasco, Italy), and/or wetted gauzes (Figure 1).

**Step 3: Customized Mesh Fitting Accuracy**

The custom-designed titanium mesh, fabricated using CAD/CAM technology, is trial-fitted to ensure a perfect match with the defect anatomy (YXoss CBR Fully Protect, ReOss, Filderstadt, Germany). One major benefit of using customized titanium mesh is the ability to pre-plan the bone volume during the diagnostic phase. This planning allows the surgeon to precisely calculate the quantity of autologous bone particles needed for grafting.

**Step 4: Creation of Bone Landmarks for Screw Fixation**

**Figure 2:**
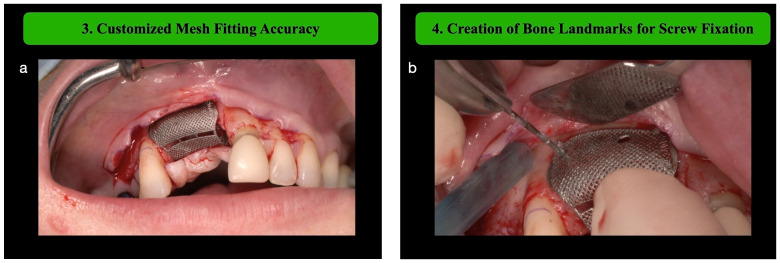
(**a**) Mesh fitting. (**b**) Bone landmark creation using a small-diameter drill (0.8).

Using a small-diameter drill (0.8 mm), landmarks are created for screw fixation. Bone landmarks are useful once the mesh is filled with the biomaterial to identify the exact position of the planned bone volume (PBV) (Figure 2).

**Step 5: Autologous Bone Harvesting**

Autologous bone is harvested from intraoral sites, such as the ramus–corpus of the homolateral mandible. A semilunar incision is performed apically 5–10 mm to the mucogingival junction, gaining access to the external oblique line. Based on the planned bone volume, the surgeon can use a safe-scraper or combine a safe-scraper with harvesting a bone block that will be particulated. Bone block harvesting can be performed using calibrated ultrasonic tips which allow osteotomies to be performed at the calculated distance from the inferior alveolar nerve, previously identified on the CBCT. The operative sequence involves four osteotomy lines, performed with two different tips. The first three osteotomies, the horizontal coronal and the two vertical ones, are performed with the straight ultrasonic tip (OT12, Mectron, Carasco, Italy), while the last osteotomy, the horizontal apical, requires an ipsilateral angled tip (OT8L-OT8R, Mectron, Carasco, Italy). It is important that each osteotomy line slightly crosses the point of connection with the others; this allows the block to be carefully collected, avoiding traumatic stress. The entity of the block is based on the PBV, taking into consideration the space between the percentages of autologous bone particles and deproteinized bovine bone matrix (DBBM), which should be at least 60:40 or even more.

**Step 6: Mesh Filling Outside the Mouth**

Gloves should be changed and cleaned using a wetted gauze [10]. The harvested bone is placed into the titanium mesh outside the mouth, mixing it with DBBM (Bio-Oss, Geistlich, Wolhusen, Switzerland). This allows for optimal bone packing and ensures a controlled, dense graft.

**Step 7: Opening Spaces in the Marrow**

Perforations are made in the recipient bone to encourage blood supply, enhance vascularization, and promote the integration of the bone graft using a small-diameter drill (0.8 mm). The recipient bone bed is prepared with multiple cortical bone perforations to activate the regional acceleratory phenomenon (RAP) and enable a better blood supply [11]. The preparation is obtained without using saline solution but at a very low speed (100/150 rpm), to avoid overheating of the bone. The dusty bone generated by the burr is maintained in situ.

**Step 8: Customized Mesh Fixation**

**Figure 3:**
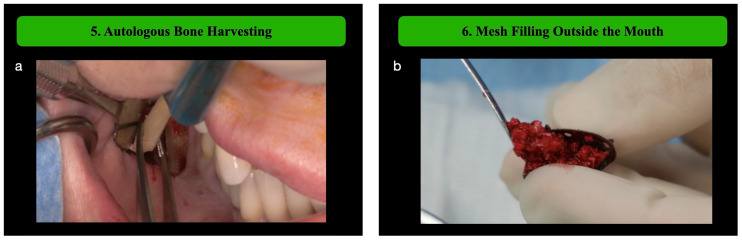

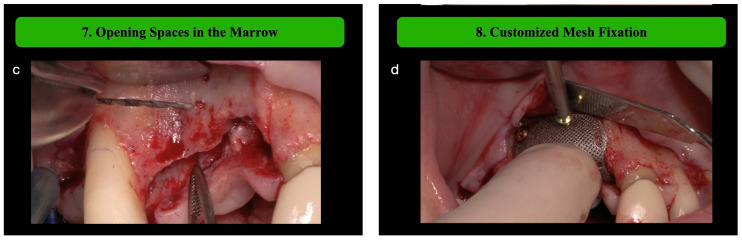
(**a**) Autologous bone harvesting using piezoelectric device. (**b**) The mesh is filled outside the mouth, using a mixture of autologous bone and deproteinized bovine bone mineral (about 80–20%, respectively). (**c**) Perforations are made in the recipient bone to encourage blood supply, enhance vascularization, and promote integration of the bone graft. The dusty bone generated by the burr is maintained in situ. (**d**) The filled titanium mesh is then fixed onto the alveolar ridge using osteosynthesis screws at pre-marked landmarks.

The filled titanium mesh is then secured onto the alveolar ridge using osteosynthesis screws (titanium screw, cross-slot, Ø 1.3 mm * 6 mm, Stoma Dentalsysteme GmbH & Co KG, Emmingen-Liptingen, Germany) at pre-marked landmarks. Mesh stability is essential for optimal bone regeneration (Figure 3).

**Step 9: Bone Pressed Inside the Mesh**

Additional graft material is gently pressed into the mesh not only to ensure full contact between the autologous bone graft and native bone, but also to avoid any kind of empty spaces inside the mesh. The biomaterial is pressed through the tracks present in the center of the mesh itself. This area has been designed as the weak region to facilitate the removal process during the second surgical phase but may be used to press the biomaterial inside the mesh using a specific compactor.

**Step 10: Bioactive Agents Application: first layer**

**Figure 4:**
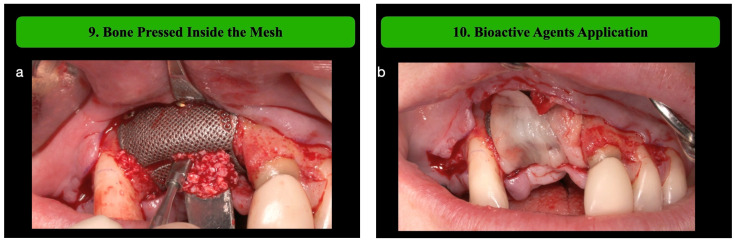
(**a**) Additional graft material is gently pressed into the mesh to avoid empty spaces under the mesh using a specific compactor. (**b**) The first CGF membranes layer is applied on the customized Ti mesh.

Bioactive agents such as concentrated growth factors (CGFs) are applied to the grafted area to enhance osteogenesis and promote bone healing (Figure 4) [12].

**Step 11: Resorbable Membrane Application**

A resorbable membrane (Bio-Gide, Geistlich, Wolhusen, Switzerland) is placed over the mesh and fixed on the buccal side with titanium tracks. The membrane acts as a barrier to prevent soft tissue from infiltrating the graft and ensuring additional stability. To assure precision in the cutting of the membrane, a template should be used.

**Step 12: Flap Elongation**

In the maxilla, flap elongation is only performed on the vestibular side, as the palatal flap cannot be moved. In the lower jaw, flap elongation is always performed also in the lingual side. On the vestibular side, flap mobilization begins with a deep partial thickness periosteal incision, followed by a more superficial incision which allow to separate the muscular structures from the mucous planes. The lingual flap management involves a stepwise approach to maximize flap release while preserving the mylohyoid muscle and minimizing complications. First, a supracrestal incision allows the elevation of the retromolar pad, integrating it into the lingual flap for an enhanced release and a reduced perforation risk. Then, the flap is gently separated from the superior fibers of the mylohyoid muscle using blunt instruments, avoiding the detachment of the muscle insertion. Finally, a semi-blunt incision in the premolar region loosens the flap further, ensuring flexibility and secure primary closure to prevent wound dehiscence [13].

**Step 13: Bioactive Agents Application: second layer**

A second layer of bioactive agents (e.g., CGFs) is applied to enhance soft tissue healing and to support the regenerative environment.

**Step 14: Flap Suture**

**Figure 5:**
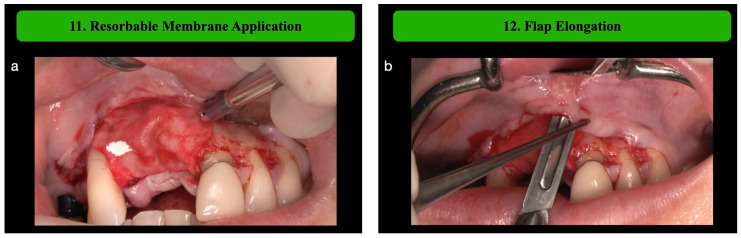

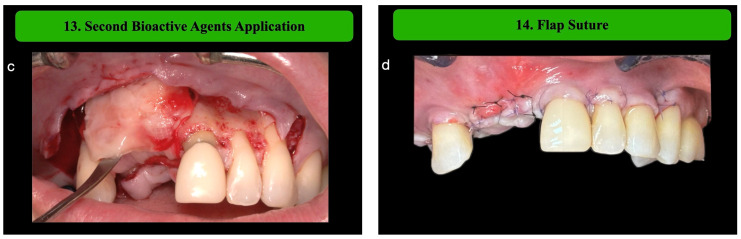
(**a**) Application and fixation of native collagen membrane on the mesh. (**b**) The flap is elongated using a 15-C blade. (**c**) A second CGF membranes layer is applied on the membrane. (**d**) Flap closure using 5-0 polyamide sutures and 6-0 polyglycolic acid sutures.

After the de-epithelialization of the anatomical papillae, performed with a 15-C blade and micro-surgical scissors, the flap is sutured using a tension-free technique, promoting optimal wound healing and preventing dehiscence over the graft. The first suture is a horizontal mattress suture in the center of the edentulous area, 5 mm apical to the primary incision line, using a 5-0 polyamide suture. The entire vestibular flap is moved coronally, using sling sutures to achieve surgical papillae stabilization over the interdental connective tissue bed, and other horizontal mattress sutures in the edentulous area [9]. This represents the first suture line. A second horizontal mattress suture line, using 6-0 polyglycolic acid suture, is performed 3 mm apical to the incision line. Lastly, a third suture line is performed using 6-0 polyglycolic acid single knots. Overall, the suture process is performed with a centripetal movement from distal to the center starting from the vertical incision (Figure 5).

**Post-operative Protocol**

Amoxicillin (875 mg) and clavulanic acid (125 mg) twice a day for 6 days, ibuprofen (600 mg) twice a day for 2 days and then as needed, and betamethasone for 5 days (dosages decreasing daily from 4 mg to 0.5 mg) are administered to the patients. Ice packs are applied intermittently for the first 2 to 3 h after surgical treatment. Patients are instructed to avoid mechanical plaque removal in the treated area for 1 week, to use chlorhexidine mouthrinse (0.12%) twice a day from the third postoperative day, and to apply chlorhexidine gel twice a day for 15 days. A soft diet is strongly recommended for three weeks.

## 3. Results

A 49-year-old Caucasian female presented with esthetic and functional demands. The clinical examination revealed a severe ridge defect in correspondence of the second sextant as a consequence of a severe peri-implantitis that affected the prognosis of the implants in that area. In fact, implants in correspondence of the right lateral and central incisors were judged as irrational to treat and were consequentially removed elsewhere. The patient’s medical history showed no systemic or local contraindications for surgical therapy.

Preoperative assessment involved CBCT imaging to evaluate bone loss and facilitate the design of the customized titanium mesh. This patient-specific scaffold is tailored to the individual anatomy. Additionally, it allows shaping the area to be augmented in the sense of backward planning for the ideal implant position and functions as a backward-planning tool, ensuring the augmented area aligns with the ideal implant position. The customized scaffold provides stable positioning for autologous bone and bone substitute material, securely anchoring them to the native bone. Using computer-aided design and manufacturing (CAD/CAM) technology, a 3D model of the bony defect is created from CBCT data. Specialized reconstruction software generates a 3D projection of the atrophied segment, allowing precise design of the scaffold. The titanium mesh is then fabricated through selective laser melting (SLM). Following the SLM process, the mesh undergoes surface treatment (blasting), ultrasonic cleaning, and is packaged in a sealed double bag for subsequent autoclaving at the surgeon’s facility.

**Figure 6:**
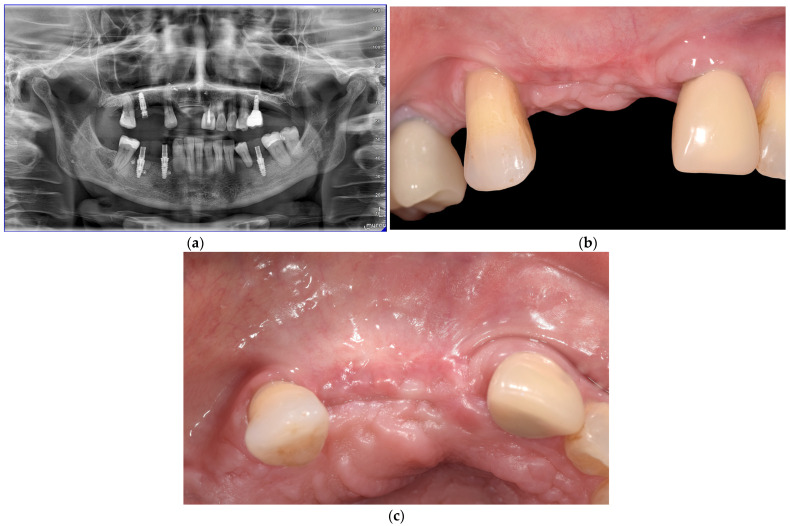
(**a**) Post-operative orthopantomography. (**b**) Frontal view of soft tissues healing after one month. (**c**) Occlusal view of soft tissues healing after one month.

Following the outlined surgical protocol, the patient underwent GBR procedure in the second sextant using the customized titanium mesh (Figure 1, Figure 2, Figure 3, Figure 4 and Figure 5). Figure 6 shows the post-operative orthopantomography and soft tissues healing, after a month. No healing complications were noted.

## 4. Discussion

The use of customized titanium mesh in guided bone regeneration offers advantages in controlling the contour and stability of the augmented site. This protocol may provide a predictable method for horizontal and vertical alveolar ridge augmentation, particularly in cases where severe atrophy limits the success of conventional implant placement [14]. Moreover, the present paper is the first one that describes a step-by-step procedure for customized titanium mesh application.

Customized titanium mesh offers a significant advantage over traditional non-resorbable membranes, primarily due to its superior mechanical properties and ability to maintain space during bone regeneration. Overall, while other non-resorbable membranes offer easier handling and removal, customized titanium meshes provide superior mechanical stability and greater customization. The added value of customized titanium meshes over other non-resorbable membranes lies in the fact that mesh design and screw fixation holes are digitally planned in advance. A recent study showed the non-inferiority of customized CAD/CAM titanium meshes when compared to reinforced polytetrafluoroethylene membranes in terms of surgical and healing complications [15]. The physical and chemical characteristics of customized Ti-meshes are crucial for the overall success of a reconstructive alveolar ridge procedure [16]. The decision of which of these materials to use should consider the defect type, surgical expertise, and clinical objectives.

The use of autologous bone remains the gold standard for bone grafting, promoting natural integration. The addition of bioactive agents such as CGF enhances the healing process, further increasing the likelihood of successful outcomes [17,18]. An innovative aspect of this protocol is the method of pressing graft material into the mesh to avoid empty spaces. By utilizing the central tracks in the mesh, the biomaterial can be compacted efficiently, ensuring the filling of the defect. This technique not only may improve the quality of the regenerated bone but may also reduce the risk of pseudoperiosteum formation [19]. Although no specific studies currently validate this approach, it aligns with known biological principles of bone healing and regeneration, where close contact between the graft material and native bone is critical for successful integration. Lastly, the application of two layers of bioactive agents, such as concentrated growth factors, represents a significant advancement in accelerating the healing process. These agents enhance both osteogenesis and soft tissue healing by stimulating cellular proliferation and differentiation at the graft site [20].

The use of customized titanium meshes provides numerous benefits compared to conventional techniques, including precise defect customization, superior mechanical stability, and predictable outcomes. These features make it particularly suitable for complex cases requiring vertical and horizontal augmentation. Future applications may include the use of resorbable custom scaffolds or incorporation of advanced bioactive agents for enhanced regeneration [21]. Despite the advantages, it is important to acknowledge the challenges associated with the use of titanium meshes. The most notable complication is the risk of mesh exposure, which can occur if the mesh is not properly fitted or if the flap closure is under tension [22]. Proper soft tissue management is critical for preventing complications and enhancing the success of bone augmentation procedures [23]. Key aspects include meticulous planning of the incision, optimal flap design, and achieving tension-free primary wound closure [18]. The precise fit of the customized mesh ensures a well-contained graft, while the use of bioactive agents accelerates the healing process and improves soft tissue integration [24]. Therefore, complications such as dehiscence may be reduced, and long-term stability may be achieved.

Moreover, it is important to consider that deviations in the accuracy of customized titanium mesh design and fabrication can arise from multiple factors, starting with the modeling of defects and the acquisition of bone contours. The accuracy and resolution of CBCT imaging are crucial and can be influenced by variables such as the system’s voxel size (0.07–0.1 mm), radiation exposure duration, and patient movement during scanning [25]. In addition, factors such as the software used for DICOM reconstruction, the selected threshold range, and processes like smoothing and digital stacking during 3D modeling can lead to unintentional bone loss on the surface contours [26]. To improve customized titanium meshes’ accuracy, higher-resolution CBCT scanning is essential for capturing the alveolar bone contours accurately. Moreover, the threshold values used during CT modeling should also be carefully selected, as they play a key role in shaping the final 3D model [24].

The division of this protocol into 14 steps acts as a useful checklist to avoid mistakes during treatment and to facilitate communication with the whole surgical team, giving the opportunity to teach the correct sequence for the entire procedure. Further studies are needed to validate the proposed surgical procedure and to assess its effectiveness. GBR with non-resorbable barriers is a highly effective surgical technique, particularly for irregular defects with a vertical component. However, this approach is associated with a relatively high complication rate, even among experienced clinicians [27]. Due to its technical complexity, GBR with non-resorbable barriers requires advanced surgical expertise and should be performed only by well-trained and experienced surgeons. The technique typically involves the use of a substantial amount of autogenous bone, which necessitates bone harvesting and further increases both procedural complexity and postoperative morbidity. Given the extended surgical duration often required, the use of intravenous sedation in combination with local anesthesia is frequently recommended to ensure patient comfort and procedural success [28].

## 5. Conclusions

The TACOS technique offers a stepwise surgical protocol for managing cases of severe alveolar atrophy using customized titanium mesh. By adhering to the outlined technique, clinicians can achieve favorable outcomes even in complex cases where bone volume is severely compromised. The combination of CAD-CAM technology, precise flap management, graft compaction techniques, and the application of bioactive agents enhance the chance of a successful outcome in most cases. While there are inherent challenges, such as the risk of mesh exposure and the potential for intraoperative inaccuracies, these can be effectively managed through careful planning and surgical technique. Further studies are needed to refine these methods and assess their long-term outcomes, particularly in relation to emerging technologies in guided bone regeneration.

## Data Availability

Data are contained within the article.
